# The non-Mendelian behavior of plant B chromosomes

**DOI:** 10.1007/s10577-022-09687-4

**Published:** 2022-04-12

**Authors:** Jianyong Chen, James A. Birchler, Andreas Houben

**Affiliations:** 1 Leibniz Institute of Plant Genetics and Crop Plant Research (IPK) Gatersleben, Corrensstrasse 3, 06466 Seeland, Germany; 2 Division of Biological Sciences, University of Missouri, Columbia, MO 65211 USA

**Keywords:** Chromosome drive, Nondisjunction, Supernumerary B chromosome, Asymmetric cell division

## Abstract

B chromosomes, also known as supernumerary chromosomes, are dispensable elements in the genome of many plants, animals, and fungi. Many B chromosomes have evolved one or more drive mechanisms to transmit themselves at a higher frequency than predicted by Mendelian genetics, and these mechanisms counteract the tendency of non-essential genetic elements to be lost over time. The frequency of Bs in a population results from a balance between their effect on host fitness and their transmission rate. Here, we will summarize the findings of the drive process of plant B chromosomes, focusing on maize and rye.

**Supplementary Information:**

The online version contains supplementary material available at 10.1007/s10577-022-09687-4.

## Introduction

The B chromosome (B) is a masterwork of evolution. This accessory chromosome was named after their distinctions from A chromosomes (As), the standard chromosomes in eukaryotes. Bs are not required for the normal growth and development of organisms, yet they are found in all eukaryotic phyla (Burt and Trivers 2006; Jones 1991; Kimura and Kayano 1961). Depending on the species, Bs may vary in behavior and DNA/chromatin composition properties (reviewed in Camacho et al. 2000; Douglas and Birchler 2017; Houben et al. 2013; Jones 1995; Birchler and Yang, 2021). Generally, it is assumed that Bs are derived from As, either from the same or from a related species (for related studies, see rye (Martis et al. 2012), *Aegilops speltoides* (Ruban et al. 2020), and maize (Blavet et al. 2021)), but follow their own evolutionary pathway (Beukeboom 1994; Camacho et al. 2000). So far, B chromosomes have been found in 2087 plant species, accounting for 2.68% of 77,958 species with a known chromosome number (D'Ambrosio et al. 2017).

Two criteria are used to distinguish Bs from the As and other special chromosomes: Bs are dispensable and do not pair with any member of the standard A chromosome complement (Jones and Rees 1982). Most Bs do not confer obvious advantages under standard growth conditions on the host plant and have no or slight effects on the host when their numbers are low. But, exceptions exist, e.g., studies of the plant pathogen *Zymoseptoria tritici* demonstrate that some Bs influence the fitness of the fungus during host infection in a cultivar-dependent manner (Habig et al. 2017). In rye, the B chromosome may contribute to heat tolerance during meiosis (Pereira et al. 2017).

**Fig. 1:**
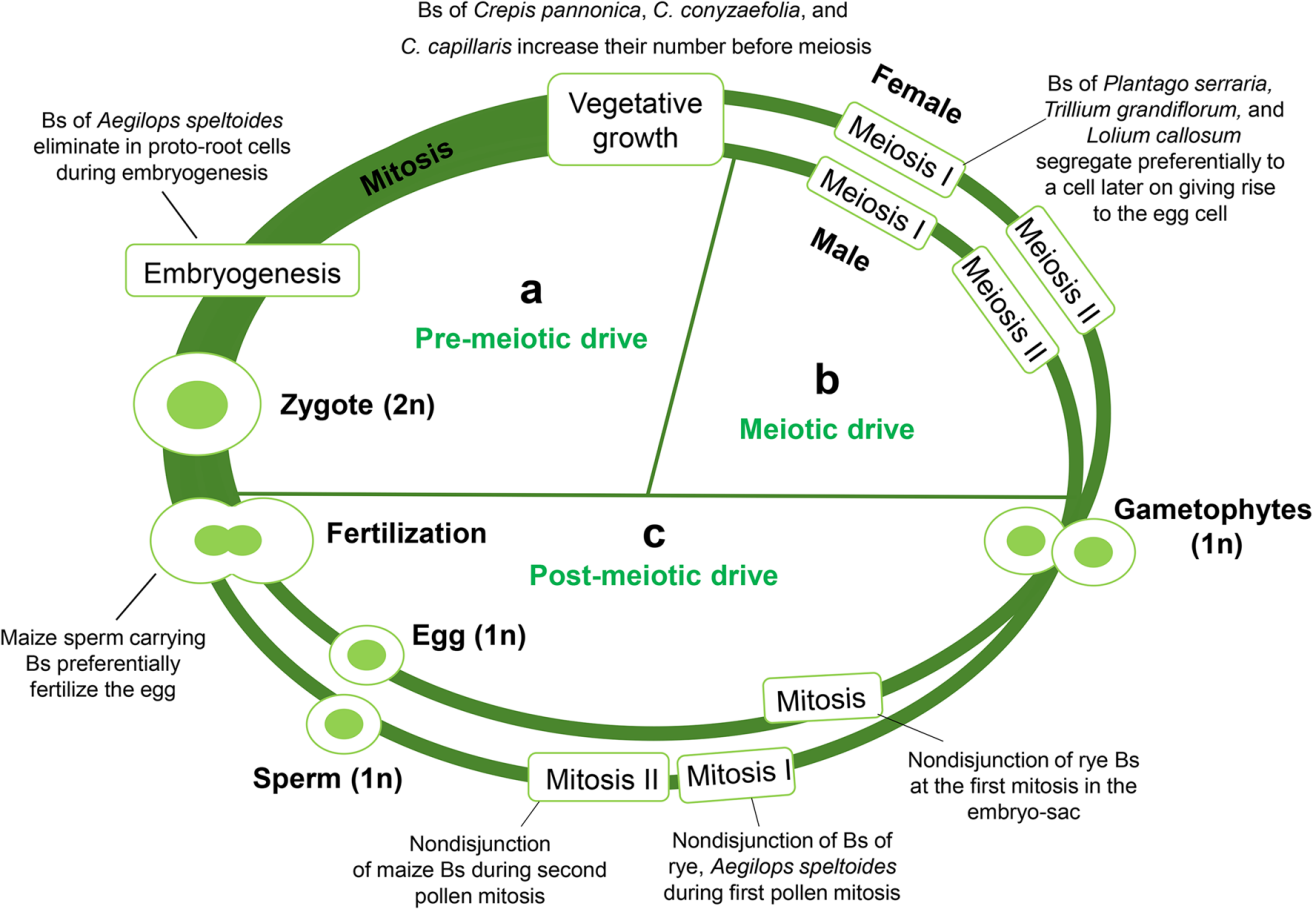
The non-Mendelian behavior of B chromosomes of different species during the live cycle of plants. The chromosome drive mechanism acts (a) pre-meiotically, (b) meiotically, and/or (c) post-meiotically. Controlled elimination of B chromosomes occurs during early embryogenesis

In contrast to Gregor Mendel’s first and second laws (the law of segregation and the law of independent assortment), the transmission of Bs in many species is greater than 0.5, a phenomenon known as genetic drive. Although the drive is one of the most important features of many B chromosomes, insights about the drive mechanism exist at the cellular level only for a few cereals. Dependent on the species, the drive mechanisms act pre-meiotically, meiotically, and/or post-meiotically (Fig. 1). Examples of species possessing a B chromosome drive are listed in Suppl. Table 1. Bs from the same genus often share a similar drive mechanism, but there are exceptions. For instance, the Bs of *Phleum phleoides* undergo nondisjunction during the first pollen division (Bosemark 1956), while the Bs of *Phleum nodosum* undergo a normal division at the first and second pollen mitosis and no accumulation of Bs was found on the male side (Bosemark 1957; Fröst 1969). On the contrary, the transmission of *P. nodosum* Bs increases through the female side (Bosemark 1957).

The maximum number of B chromosomes that can accumulate in an individual varies among species (e.g., maize and rye could possess up to 34 and 6 Bs, respectively) and likely depends on the balance between drive efficiency and adverse effects like reduced fertility and vigor caused by the B. However, Bs without drive also exist. According to the transmission data of Bs from about 70 species, only about 60% of Bs can drive (Jones 1995). Bs without drive likely counteract their dispensable nature by providing beneficial features to the host species. For instance, in *Allium schoenoprasum*, the germination rate and survival of B-containing individuals was higher than that of individuals without Bs in drought conditions (Plowman and Bougourd 1994). Supernumerary chromosomes of the blast fungus *Magnaporthe oryzae* contribute to adaptive evolution by carrying a virulence-related gene and participating in genome rearrangement (Langner et al. 2021). B chromosome polymorphism could be interpreted as a dynamic system in which the frequency of Bs in a population continually shifts due to an “arms race” between the standard A and supernumerary B chromosomes (Camacho et al. 2000).

## Nondisjunction in favor of a chromosome

Nondisjunction of sister chromatids is likely a key component of the B chromosome drive in many species. Nondisjunction occurs when both sister chromatids migrate to the same daughter cell during division. This can happen if the sister chromosomes are held together post replication by DNA-DNA topological entanglement and the cohesion complex. Lagging chromosomes are also known to arise from error-prone kinetochore-microtubule interactions (reviewed by Kamenz and Hauf 2017). While nondisjunction of A chromosomes causes aneuploid and often results in cell death or genetic instability, controlled nondisjunction of Bs at a defined developmental stage, e.g., the first pollen grain mitosis, allows Bs to accumulate in the generative nuclei. Notably, despite this lagging of Bs, the cell cycle progresses, and no cell death occurs. Whether the intrinsic mitotic spindle assembly checkpoint is impaired or Bs escape the checkpoint control remains unknown.

## Pre-meiotic and meiotic drive of Bs

Accumulation of Bs before meiosis has been reported in only a few plant species, and it is more common in animals (Austin et al. 2009). In *Crepis pannonica*, *Crepis conyzaefolia*, and *Crepis capillaris*, a higher number of Bs in pollen mother cells (PMCs) were found compared with their number in root meristems (Fröst 1964; Fröst and Östergren 1959; Rutishauser and Rothlisberger 1966). Nondisjunction of B sister chromatids occurs during inflorescence development in a genotype-dependent manner (Fig. 1(a)). As a result, the inflorescences of many *C. capillaris* plants carrying Bs are mosaics with PMCs having varying numbers of Bs (Parker et al. 1989; Rutishauser and Rothlisberger 1966).

The asymmetry of female meiosis and cell division to produce the egg cell in some plants and vertebrates provides an opportunity for genetic drive. Only one product of meiosis becomes an egg nucleus, and the other three products do not contribute genetically to the next generation. This sets the stage for mechanisms that cause chromosomes to preferentially end up in the egg nucleus as opposed to the other products of meiosis (Fig. 1(b)). In *Plantago serraria*, *Trillium grandiflorum*, and *Lilium callosum*, Bs segregate preferentially to a cell later on, giving rise to the egg cell during meiosis. During meiosis I (MI), the majority of the Bs were seen lying outside the MI plate and on the micropylar side, the side which would give rise to the egg cell (Kayano 1957). No mechanism of numerical increase of Bs to the next generation was found on the male side in the same species.

Although not involving a B chromosome, a well-studied mechanism of meiotic drive represents the abnormal A chromosome 10 (Ab10) of maize (Rhoades 1942; Longley 1945). With the aid of a kinesin-14 motor located on Ab10, large blocks of heterochromatin called knobs that are variably present on many corn chromosomes moves ahead of the rest of the genome to the micropylar direction at the anaphase of MI and MII. This causes chromosomes with large knobs, including Ab10, to be positioned in the lower cell, which will go on to form an egg (Dawe et al. 2018). Different from female meiosis, male meiosis produces four spores (tetrads), which all lead to gametes that compete to fertilize an egg cell and drive is not observed for Ab10 through the male. The drive of maize Bs and Ab10 occurs at different life cycle stages, one in meiosis (Ab10) and the other at the second pollen mitosis, so the drive mechanisms are different. But, Ab10 is another example of a dispensable genetic element that persists in maize populations via genetic drive.

Different from female meiosis, male meiosis produces four spores (tetrads), which all lead to gametes that have equal opportunity to fertilize an egg cell. Unpaired chromosomes such as those present in aneuploids are often lost due to their irregular behavior at meiosis I. Because only a single B chromosome is frequently present in individuals within populations that carry B chromosomes, B chromosomes may benefit from mechanisms or features that enable them to transit meiosis as a univalent chromosome. In order to reduce its meiotic loss, the rye B increases its ability to form B bivalents at the metaphase of MI (Jiménez et al. 1997). However, different from the drive, rye Bs moderate their polymorphic transmission rates to reach the gain/loss balance since too many Bs would affect the fitness of the host plants (Puertas et al. 1998). The maize B has the property to help its meiotic transmission as a univalent (Carlson and Roseman, 1992). This property is dependent on regions of the B chromosome (Carlson and Roseman, 1992) itself as well as the genetic background (Gonzalez-Sanchez et al. 2007). The loss of maize B univalent in a low transmission line is due to the misorientation of the Bs during metaphase-anaphase I; on the other hand, the B univalent in the high transmission line is always correctly oriented (González-Sánchez et al. 2007).

## Post-meiotic drive of Bs

**Fig. 2:**
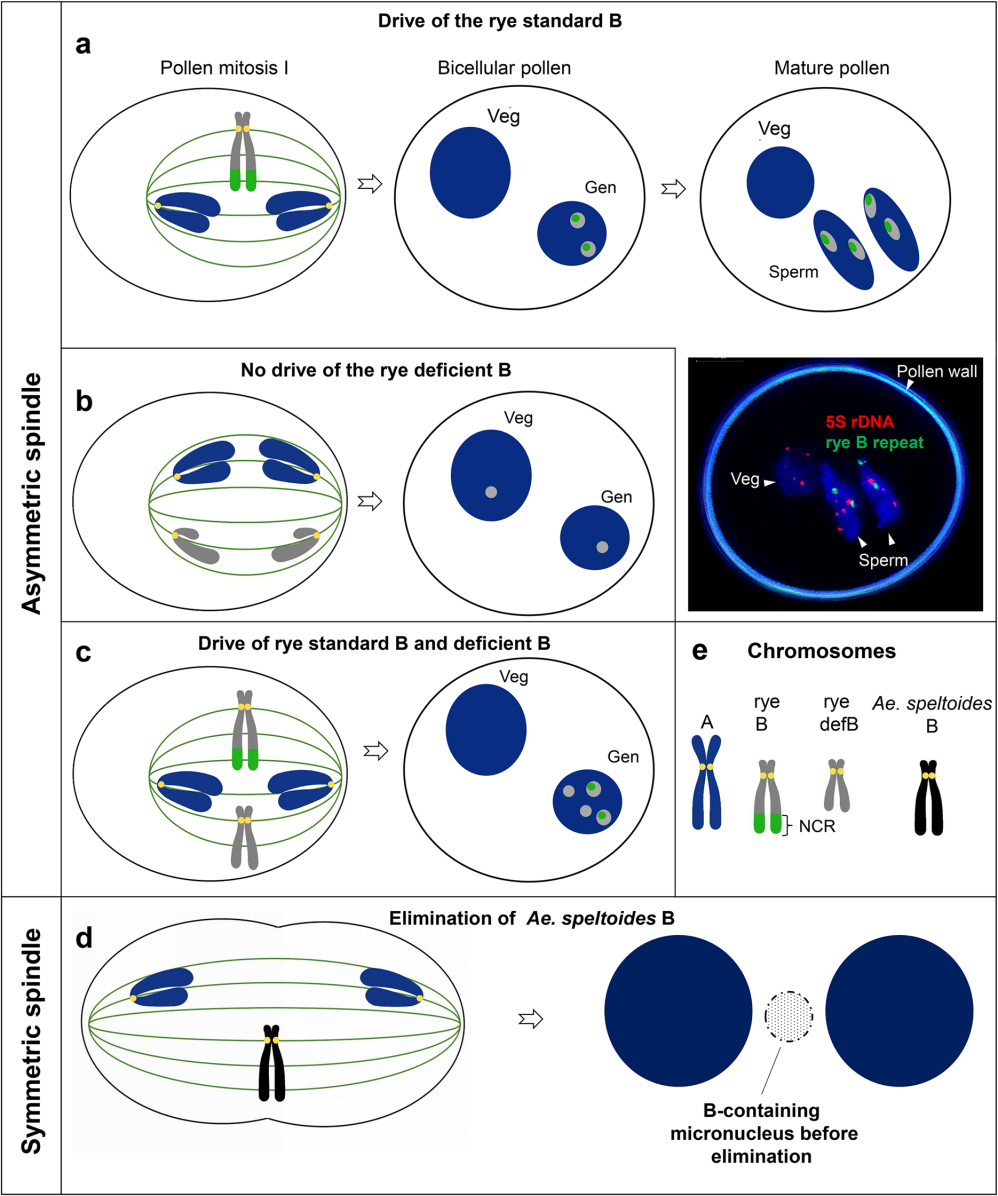
The segregation behavior of rye and *Ae. speltoides* B chromosomes. **a**–**c** Drive of the rye B occurs during the first pollen grain mitosis. Veg, vegetative nucleus; Gen, generative nucleus. **a** Standard B behavior during pollen development. Mature pollen of rye with Bs after FISH with B-repeat (in green) and A chromosome–specific 5S rDNA (in red). Bs accumulate in sperm nuclei due to nondisjunction and asymmetric spindle formation. **b** No drive occurs if the nondisjunction control region (NCR) of the B is missing. **c** Standard B with a complete NCR and deficient B variants. Drive of both B variants occurs (Endo et al. 2008). NCR acts in trans. **d** Nondisjunction of the *Ae. speltoides* B in proto-root cells during early embryogenesis results in micronucleation and complete elimination. In contrast to B chromosome drive, a symmetrical cell division occurs as part of the chromosome elimination process. **e** Different types of chromosomes. The rye B’s NCR is shown in green

The post-meiotic accumulation of Bs is the best-analyzed drive process and is frequent in plants, where the formation of mature pollen involves two post-meiotic divisions that result in generative and vegetative nuclei (Fig. 1(c)). In rye and *A. speltoides* during the first pollen mitosis, Bs undergo nondisjunction and then will be included in the generative nucleus or remain lagging so that most vegetative nuclei receive A chromosomes only (Fig. 2a). A quantitative flow cytometric approach revealed that independent of the number of Bs present in the mother plant, Bs accumulate in the generative nuclei to > 93% in *Ae. speltoides* (Wu et al. 2019). Similarly, in rye, the nondisjunction of Bs is a highly efficient process in all kinds of populations, from Turkey (93%), Iran (92%), South Korea (93%), Japan (96%) (Niwa and Sakamoto 1995), and China (88%) (Niwa and Sakamoto 1996). In weedy rye (*Secale segetale*) from Pakistan, a 95% B nondisjunction frequency was found (Niwa and Sakamoto 1996). At the second pollen mitosis, B sister chromatids normally divide like standard chromosomes, and each sperm nucleus contains the same number of Bs (Fig. 2a).

Nondisjunction is not common on the female side in those plants whose Bs exhibit nondisjunction in pollen grains. For example, crossing results indicated no drive of Bs of *Anthoxanthum aristatum* (Östergren 1947), *Festuca pratensis* (Bosemark 1954), *P. phleoides* (Bosemark 1956), and *Ae. speltoides* (Mendelson and Zohary 1972) occurring on the female side. On the other hand, the B chromosome of rye could drive during the development of female gametophytes. The nondisjunction of rye Bs was observed at the first mitosis in the embryo sac (Håkansson 1948).

The drive of the rye B works equally well when the B was introduced as an additional chromosome into *Secale vavilovii* (Puertas et al. 1985), hexaploid wheat (Endo et al. 2008; Lindström 1965; Müntzing 1970; Niwa et al. 1997), or hypo-pentaploid *Triticale* (Kishikawa and Suzuki 1982). Thus, the rye B controls the nondisjunction process by itself (Matthews and Jones 1983; Romera et al. 1991).

The heterochromatic end of the rye long B arm is involved in the control of nondisjunction. B chromosomes lacking the so-called nondisjunction control region (NCR) undergo normal disjunction at the first pollen mitosis (Fig. 2b). This region acts in trans because nondisjunction occurs for the NCR-deficient B (defB) if a standard B (Lima-de-Faria 1962) or the NCR of the long arm of the B (Endo et al. 2008) is present in the same cell containing a NCR-deficient B (Fig. 2c). Thus, defB carries the *cis*-acting element(s) responsible for nondisjunction. Although the Giemsa banding–positive NCR is a hot spot of B-specific satellite DNAs, containing at least 8 different satellite repeats (Blunden et al. 1993; Carchilan et al. 2007; Klemme et al. 2013; Sandery et al. 1990; Wu et al. 2019), this region also encodes transcriptionally active protein-coding genes (Chen, Boudichevskaia et al. unpublished).

The NCR replicates later than the rest of the entire genome (Klemme et al. 2013; Lima-de-Faria and Jaworska 1972). The peculiarity of the NCR lies in the fact that, contrary to the Giemsa-positive subtelomeric heterochromatic regions of As, this domain is simultaneously marked by trimethylated histone H3K4 and trimethylated H3K27 (Carchilan et al. 2007), an unusual combination of apparently conflicting post-translational histone modifications. Unknown is whether nonrepressive (H3K4me) and repressive (H3K27me) histone modifications coexist within the same nucleosome or whether they occupy alternate nucleosomes.

Does a dysfunctional centromere cause or contribute to the mechanisms of nondisjunction of Bs? In rye and *Ae. speltoides*, no major differences in the CENH3 (CENPA) signal size were found between A and B centromeres and the interaction between B centromeres and tubulin fibers was observed (Banaei-Moghaddam et al. 2012; Wu et al. 2019). Thus, a different centromere activity of Bs might be excluded. An important hint regarding the mechanism of chromosome drive comes from the finding that the microtubule spindle of both species is asymmetrical during the first pollen mitosis, in accordance with previous studies in other species (Banaei-Moghaddam et al. 2012; Borg et al. 2009; Wu et al. 2019). It is likely that the inclusion of lagging Bs into the generative nucleus is caused by the fact that the equatorial plate is nearer to the generative pole. Spindle asymmetry as a component of the drive process has also been suggested for the Bs of the lily *L. callosum* (Kimura and Kayano 1961), the Asteraceae *C. capillaris* (Rutishauser and Rothlisberger 1966), and the grasshopper *Myrmeleotettix maculatus* (Robinson and Hewitt 1976). But, also standard meiotic mouse A chromosomes with a stronger centromere harness the spindle asymmetry to drive (Akera et al. 2017). Notably, contrary to drive, the targeted loss of Bs is also caused by nondisjunction. However, in contrast to drive, a symmetrical cell division occurs as part of the chromosome elimination process. In *Ae. speltoides*, the unresolved sister chromatid cohesion of Bs leads to nondisjunction, micronucleation, and subsequent elimination during early embryogenesis of proto-root cells (Ruban et al. 2020) (Fig. 2d). Not only in plants but also in animals, nondisjunction of specific chromosomes leads to programmed DNA elimination. The extended cohesion between sister chromatids at the distal end at anaphase results in a partial loss of the sea lamprey (*Petromyzon marinus*) genome during early embryogenesis (Smith et al.^ 2021^; Timoshevskiy et al. 2016). A programmed chromosome elimination process also occurs in *Sciara* species; paternal X chromosomes undergo elimination during embryonic development since nondisjunction of them at the distal ends gives rise to their retardation after anaphase segregation (Escribá and Goday 2013).

It is possible that in *Ae. speltoides* and rye, the cohesion between B sister chromatids during first pollen mitosis is stronger than the microtubule traction force required to divide chromatids. But why does the cohesion differ between A and B chromatids? A B chromosome–specific composition of (peri)centromere sequences was observed for the Bs of rye (Banaei-Moghaddam et al. 2012), *Ae. speltoides* (Wu et al. 2019), maize (Jin et al. 2005; Lamb et al. 2005), *F. pratensis* (Ebrahimzadegan et al. 2019), the daisy *Brachycome dichromosomatica* (Leach et al. 1995), and the grasshopper *Xyleus discoideus angulatus* (Bernardino et al. 2017). It is likely that the B-specific (peri)centromere sequence composition is functionally involved in the drive of Bs. In addition, heterochromatin, checkpoint control, release of cohesion, or related yet unidentified proteins may differ between A and B chromosomes and result in different segregation dynamics of Bs. It is tempting to speculate that the extended cohesion of B sister chromatids is also part of their behavior during meiosis if occurring as a univalent. Unlike As, rye B univalents split sister chromatids less frequently at anaphase I (Manzanero et al. 2000).

## Nondisjunction of Bs at the second pollen mitosis and preferential fertilization

**Fig. 3:**
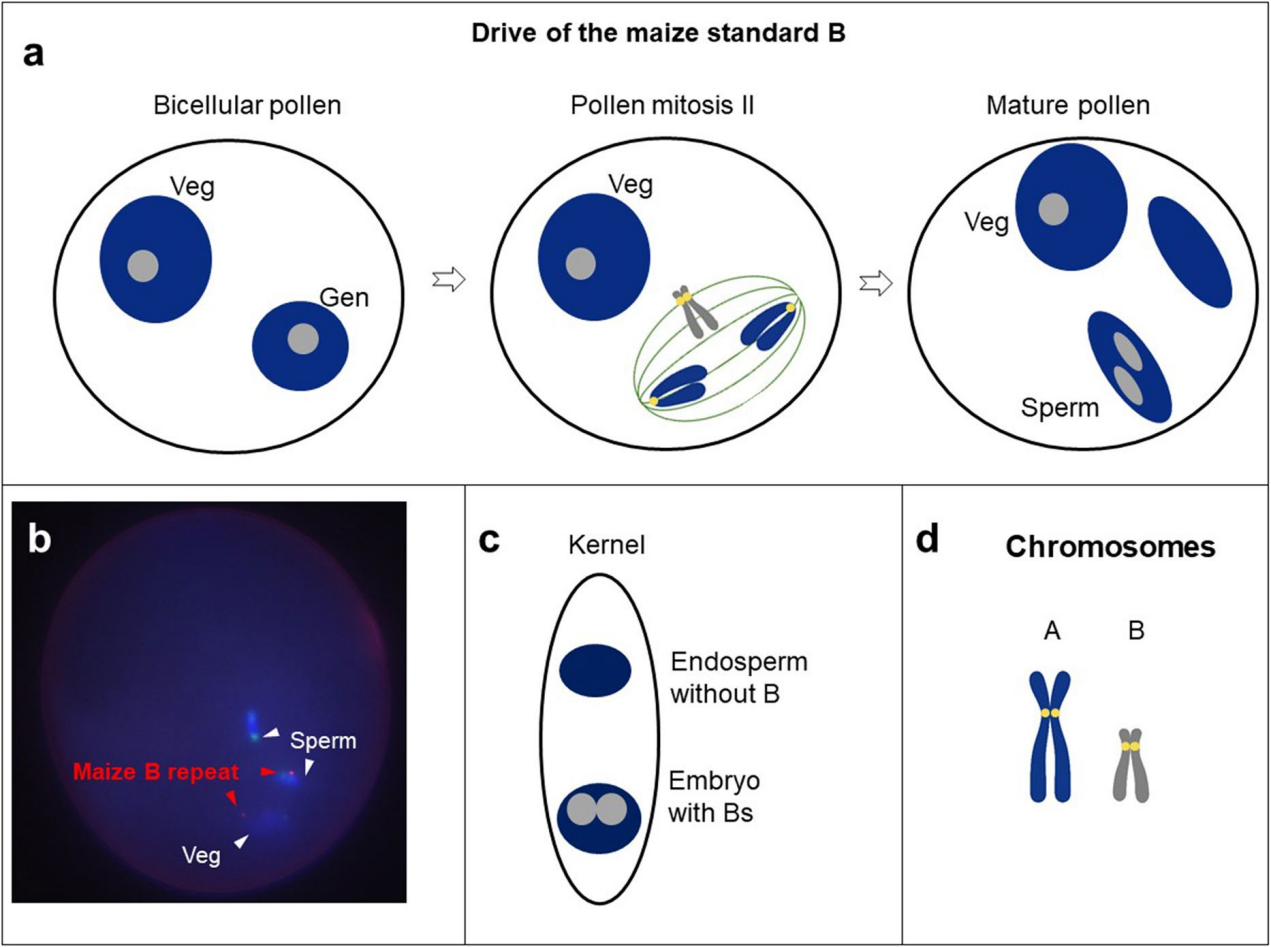
Drive of the maize B chromosome. **a** At the first pollen mitosis, the maize B behaves like As. Consequently, both vegetative (Veg) and generative nuclei (Gen) contain Bs. Nondisjunction of the B occurs at second pollen mitosis, and one sperm nucleus accumulates the Bs. **b** Mature pollen of a +2B maize plant after FISH using a B-specific repeat (in red). **c** At the fertilization process, the sperm with Bs preferentially fertilizes the egg, which results in an embryo with Bs and endosperm without Bs. **d** Different types of chromosomes

Maize, which belongs to the subfamily *Panicoideae*, evolved a different B drive mechanism. Bs of maize will undergo nondisjunction during the second pollen division (Roman 1947). In addition, the nondisjunction of maize Bs occurs during the first pollen division, but its frequency is very low (Rusche et al. 1997). Nondisjunction results in one sperm nucleus containing duplicate Bs and a sperm nucleus without Bs (Fig. 3a, b). Thus, at the subsequent double fertilization, the sperm nuclei with Bs will fertilize with the egg and the other one without Bs will fuse with two polar nuclei with the preference for the egg being about 67% in most lines (Roman 1948) (Fig. 3c). In short, the drive process of maize Bs takes two steps: nondisjunction and preferential fertilization. Interestingly, some lines of maize have random fertilization with regard to the presence/absence of a B chromosome (Carlson 1969) or even a reversal of preferential fertilization with the B-containing sperm joining with the polar nuclei more often than with the egg (Carlson 1999).

The maize B chromosome has a B chromosome–specific repeat that is in and around the centromere (Alfenito and Birchler 1993; Jin et al. 2005; Blavet et al. 2021). Sequence analysis indicates that it is the only unique sequence in minichromosomes derived from the maize B that are still capable of nondisjunction, thus implicating it as the target for mediating the process. The repeat spans several megabases and is heterogeneous but generally has a segment with homology to heterochromatic knobs (Alfenito and Birchler 1993; Hsu et al. 2003) as well as degenerate telomere repeat arrays.

The nondisjunction of the maize B centromere requires trans-acting factors elsewhere on the chromosome (Roman 1947; Ward 1973a; Lin 1978; Auger and Birchler 2002). One of these is located near the very distal tip of the long arm. The sequence information locates the responsible region to one with 34 predicted protein-encoding genes (Blavet et al. 2021), although other sources of a trans-factor are possible from this segment.

In addition to the nondisjunction and preferential fertilization, the maize B has other properties that help perpetuate it. It has long been known that the B chromosome will foster increased recombination in the A chromosomes, particularly on the male side and apparently in pericentromeric heterochromatin (Ayonoadu and Rees 1968; Rhoades 1968; Hanson 1969; Nel 1973; Ward 1973b; Robertson 1984; Carlson et al. 1993). The presumed reason for this stimulation is that it conditions recombination in its own highly heterochromatic regions to facilitate faithful separation in meiosis I (Carlson 1994). It is notable that the increase is greater on the male side, which immediately precedes the aspects of the drive mechanism involving nondisjunction at the second pollen mitosis.

Another property of the maize B that aids its perpetuation is its stabilization as a univalent in meiosis (Carlson and Roseman 1992; Gonzalez-Sanchez et al. 2007). The maize B chromosome can find itself alone in meiosis, which with most chromosomes will lead to a high frequency of loss. This process also appears to involve the B-specific repeat in the centromeric region (Gonzalez-Sanchez et al. 2007), which shows a precocious attachment to the meiotic spindle before the normal centromere analogous to abnormal chromosome 10, described above. There is, nevertheless, a clear distinction because the maize B chromosome does not induce the heterochromatic knobs on the A chromosomes to show preferential segregation.

The maize B chromosome and the heterochromatic knobs have an apparent antagonism, given that there is a negative correlation between the presence of knobs and B chromosomes in Native American collections of maize (Longley 1938; Bianchi et al. 1963). Indeed, in a line described by Rhoades et al. (1967) and Rhoades and Dempsey (1972), the presence of B chromosomes will cause the knobs in the A chromosome to remain adhered at the second pollen mitosis—the same mitosis at which the B centromere nondisjoins. The consequence is that the knobbed chromosomes break at this mitosis at a high frequency. Because the maize B chromosome and knobs are both late replicating in S phase, the hypothesis put forward to explain this interaction was that the high-loss line interacts with the B chromosome to cause an even more delayed replication of both, resulting in the chromosomal breakage.

The control of nondisjunction, increased recombination of the A complement, univalent stability, and interaction with knobs all appear to be affected by genes encoded on the maize B chromosome, and indeed, various regions have been attributed to these functions (Birchler and Yang 2021). The sequence of the maize B chromosome revealed 758 predicted protein encoding genes, many of which are expressed (Huang et al. 2016), but did not reveal any synteny with a potential progenitor region in the A chromosomes. Instead, the sequence revealed that the paralogues shared with the A chromosomes were dispersed and had widely different divergence times (Blavet et al. 2021). Thus, it appears that the maize B chromosome has been in the evolutionary lineage for millions of years during which the initial genes have deteriorated beyond recognition. The current gene repertoire is likely to have resulted from a continuous transposition of genes from the A complement over millions of years. Many predicted genes on the maize B show evidence of relaxed purifying selection. However, some of the genes likely have evolved to perform functions for the perpetuation of the B despite its nonvital nature.

## Outlook

B chromosome sequences are notoriously difficult to assembly due to their complex mosaic composition of A chromosome–derived and organelle-derived DNA fragments and their high repeat composition (Blavet et al. 2021). However, the recent development of accurate long-read sequencing by PacBio circular consensus sequencing (CCS), HiC, optical mapping, and nanopore sequencing greatly improved the sequence assembly of large genome species at the chromosome scale, and the first nearly complete plant B chromosome sequence became available (Blavet et al. 2021). The accessibility of genomic B sequences in combination with tissue-specific transcriptome data of B chromosome mutants with and without drive will greatly speed up the quest for genes controlling the drive of B chromosomes. A detailed understanding of the molecular mechanism underlying the tissue and chromosome type–specific drive of Bs will provide clues about the process of chromosome nondisjunction, which is a major cause of genetic diseases across species.

## Supplementary information

Supplementary Table 1 Mechanisms of B chromosome accumulation in plants (based on Jones and Rees 1982 and recent publications). (DOCX 29 kb)

## Abbreviations

A: A chromosome
Ab10: abnormal A chromosome 10
B: B chromosome
CENH3: centromere-specific histone H3
Gen: generative
NCR: nondisjunction control region
PMC: pollen mother cell
Veg: vegetative
