# Supplementary material for: The non-Mendelian behavior of plant B chromosomes
Source: Chromosome Res. 2022 Apr 12;30(2-3):229–39. doi: 10.1007/s10577-022-09687-4 (PMC9508019; doi:10.1007/s10577-022-09687-4)
Supplement: Supplementary file 1 — Mechanisms of B chromosome accumulation in plants (based on Jones and Rees 1982 and recent publications). (DOCX 29 kb) [file 10577_2022_9687_MOESM1_ESM.docx]

Table S1 Mechanisms of B chromosome accumulation in plants (Modified from Jones and Rees 1982 and recent publications)

| **Type** | **Chromosome behavior** | **Species** | **Family** | **Subfamily** | **Absence** | **Reference** |
| --- | --- | --- | --- | --- | --- | --- |
| Pre-meiotic drive | - | *Crepis pannonica* | Asteraceae | - | - | Fröst and Östergren 1959; Fröst 1964 |
|  | - | *Crepis conyzaefolia* | Asteraceae | - | - |  |
|  | Somatic non-disjunction in vegatative tissues | *Crepis capillaris* (Swiss accession) | Asteraceae | - | - | Rutishauser and Rothlisberger 1966; Parker et al. 1989 |
| Meiotic drive | Preferential meiotic segregation in EMCs | *Phleum nodosum* | Gramineae | Pooideae | - | Bosemark 1957 |
|  |  | *Cochlearia pyrenaica* | Brassicaceae | - | - | Gill 1971 |
|  |  | *Lilium callosum* | Liliaceae | Lilioideae | - | Kayano 1957 |
|  |  | *Plantago serraria* | Plantaginaceae | - | - | Fröst 1959 |
|  |  | *Tradescantia virginiana* | Commelinaceae |  | - | Vosa 1962 |
|  |  | *Trillium grandiflorum* | Melanthiaceae | - | - | Rutishauser 1956 |
| Post-meiotic drive | Non-disjunction at the first pollen mitosis | *Brachycome dichromosomatica* | Asteraceae | Asteroideae | - | Carter 1978 |
|  |  | *Haplopappus gracilis* |  |  | in roots | Östergren and Fröst 1962; Pritchard 1968 |
|  | Preferential distribution during pollen mitosis | *Xanthisma texanum* | Asteraceae | - | Totally absent in primary roots and partially in adventitious roots and tapetal tissue | Berger and Witkus 1954; Semple 1972, 1976; Semple and Chmielewski 1989 |
|  | Non-disjunction at the first pollen mitosis | *Holcus lanatus* | Gramineae | Pooideae | - | Bosemark 1957 |
|  |  | *Phleum phleoides* |  |  | - | Bosemark 1956 |
|  |  | *Anthoxanthum aristutum* |  |  | - | Östergren 1947 |
|  |  | *Alopecurus pratensis* |  |  | - | Bosemark 1957 |
|  |  | *Briza media* |  |  | - | Bosemark 1957 |
|  |  | *Dactylis glomerata* |  |  | Somatic instability at the stage of tiller differentiation | Puteyevsky and Zohary 1971 |
|  |  | *Deschampsia bottnica* |  |  | - | Albers 1972 |
|  |  | *Deschampsia caespitosa* |  |  | - |  |
|  |  | *Deschampsia wibeliana* |  |  | - |  |
|  |  | *Festuca pratensis* |  |  | - | Bosemark 1954 |
|  |  | *Festuca arundinacea* |  |  | - | Bosemark 1957 |
|  | Non-disjunction at the first pollen mitosis; Drive during the egg cell development | *Aegilops speltoides* | Gramineae | Pooideae | In roots | Mendelson and Zohary 1972; Wu et al. 2019; Ruban et al. 2020 |
|  |  | *Aegilops mutica* |  |  | In roots | Mochizuki 1957; Ohta 1996 |
|  | Non-disjunction at the first pollen mitosis; Non-disjunction at the first egg cell mitosis | *Secale cereale* | Gramineae | Pooideae | - | Hasegawa 1934; Håkansson 1948a; Banaei-Moghaddam et al. 2012; Endo et al. 2008; Gonzalez-Sanchez et al. 2014; Jimenez et al. 1998, 2000; Jimenez et al. 1997; Jimenez et al. 1994; Neves et al. 1992; Ortiz et al. 1996; Puertas et al. 1998a, 2000; Puertas et al. 1998b; Puertas et al. 1990; Romera et al. 1991 |
|  | - | *Sorghum purpureosericeum* | Gramineae | Panicoideae | In all tissues except anthers and ovary | Darlington and Thomas 1941; [Karafiátová](javascript:;) et al. 2021 |
|  | Non-disjunction at the second pollen mitosis and preferential fertilization with egg cells | *Zea mays* | Gramineae | Panicoideae | - | Blavet et al. 2021; Carlson 1988; Chiavarino et al. 1998; Han et al. 2007a; Han et al. 2007b; Masonbrink and Birchler 2010; Roman 1947; Tseng et al. 2017; Su et al. 2018 |
| No drive | Non-disjunction in some differentiating anthers | *Allium schoenoprasum* | Amaryllidaceae | Allioideae | - | Bougourd and Parker 1979 |
|  | - | *Centaurea scabiosa* | Asteraceae | Carduoideae | - | Fröst 1956, 1957 |
|  | - | *Poa alpina* | Gramineae | Pooideae | In adventitious roots | Håkansson 1948b; Müntzing 1948 |
|  | Chromosome number is variable in a plant | *Ranunculus acris* | Ranunculaceae | Ranunculoideae | - | Fröst 1969 |
|  |  | *Ranunculus ficaria* | Ranunculaceae | Ranunculoideae | - | McLeish 1954 |

Note: “-” indicates unknown

**References**

Albers F (1972) Cytotaxonomie und B-Chromosomen bei *Deschampsia caespitosa* (1.) PB und verwandten Arten. Beiträge zur Biologie der Pflanzen

Banaei-Moghaddam AM, Schubert V, Kumke K, Weibeta O, Klemme S, Nagaki K, Macas J, Gonzalez-Sanchez M, Heredia V, Gomez-Revilla D, Gonzalez-Garcia M, Vega JM, Puertas MJ, Houben A (2012) Nondisjunction in favor of a chromosome: The mechanism of rye B chromosome drive during pollen mitosis. Plant Cell 24:4124-4134

Berger C, Witkus E (1954) The cytology of *Xanthisma texanum* DCI differences in the chromosome number of root and shoot. Bulletin of the Torrey Botanical Club 81:489-491

Blavet N, Yang H, Su H, Solansky P, Douglas RN, Karafiatova M, Simkova L, Zhang J, Liu Y, Hou J, Shi X, Chen C, El-Walid M, McCaw ME, Albert PS, Gao Z, Zhao C, Ben-Zvi G, Glick L, Kol G, Shi J, Vrana J, Simkova H, Lamb JC, Newton K, Dawe RK, Dolezel J, Ji T, Baruch K, Cheng J, Han F, Birchler JA, Bartos J (2021) Sequence of the supernumerary B chromosome of maize provides insight into its drive mechanism and evolution. Proc Natl Acad Sci USA 118

Bosemark NO (1954) On accessory chromosomes In *Festuca* *pratensis*: I. Cytological investigations. Hereditas 40:346-376

Bosemark NO (1956) Cytogenetics of accessory chromosomes in *Phleum* *phleoides*. Hereditas 42:443-466

Bosemark NO (1957) Further studies on accessory chromosomes in grasses. Hereditas 43:236-297

Bougourd SM, Parker JS (1979) The B-chromosome system of *Allium* *schoenoprasum*. Chromosoma 75:369-383

Carlson WR (1988) B chromosomes as a model system for nondisjunction. In Vig BK, Sandberg AA, eds Aneuploidy, Part B: In-duction and Test Systems New York, Alan R Liss:199-207

Carter C (1978) The cytology of *Brachycome*. Chromosoma 67:109-121

Chiavarino AM, Rosato M, Rosi P, Poggio L, Naranjo CA (1998) Localization of the genes controlling B chromosome transmission rate in maize (*Zea mays* ssp. mays, Poaceae). Am J Bot 85:1581-1585

Darlington CD, Thomas P (1941) Morbid mitosis and the activity of inert chromosomes in Sorghum. Proceedings of the Royal Society of London Series B-Biological Sciences 130:127-150

Endo TR, Nasuda S, Jones N, Dou Q, Akahori A, Wakimoto M, Tanaka H, Niwa K, Tsujimoto H (2008) Dissection of rye B chromosomes, and nondisjunction properties of the dissected segments in a common wheat background. Genes Genet Syst 83:23-30

Fröst S (1956) The cytological behaviour of accessory chromosomes in *Centaurea* *scabiosa*. Hereditas 42:415-430

Fröst S (1957) The inheritance of the accessory chromosomes in *Centaurea* *scabiosa*. Hereditas 43:403-422

Fröst S (1964) Further studies of accessory chromosomes in *Crepis* *conyzaefolia*. Hereditas 52:237-239

Fröst S (1969) The inheritance of accessory chromosomes in plants, especially in *Ranunculus* *acris* and *Phleum nodosum*. Hereditas 61:317-326

Fröst S, Östergren G (1959) *Crepis pannonica* and *Crepis conyzaefolia*‐Two more species having accessory chromosomes. Hereditas 45:211-214

Fröst, S. (1959). The cytological behaviour and mode of transmission of accessory chromosomes in *Plantago* *serraria*. Hereditas, 45(2-3), 191-210.

Gill J (1971) The cytology and transmission of accessory chromosomes in *Cochlearia* *pyrenaica* DC.(Cruciferae). Caryologia 24:173-181

Gonzalez-Sanchez M, Heredia V, Diez M, Puertas MJ (2014) Rye B chromosomes influence the dynamics of histone H3 methylation during microgametogenesis. Cytogenet Genome Res 143:189-199

Håkansson A (1948a) Behaviour of accessory rye chromosomes in the embryo‐sac. Hereditas 34:35-59

Håkansson A (1948b) Embryology of *Poa* *alpina* plants with accessory chromosomes. Hereditas 34:233-247

Han F, Gao Z, Yu W, Birchler JA (2007a) Minichromosome analysis of chromosome pairing, disjunction, and sister chromatid cohesion in maize. Plant Cell 19:3853-3863

Han F, Lamb JC, Yu W, Gao Z, Birchler JA (2007b) Centromere function and nondisjunction are independent components of the maize B chromosome accumulation mechanism. Plant Cell 19:524-533

Hasegawa N (1934) A cytological study on 8-chromosome rye. Cytologia 6:68-77

Jimenez G, Manzanero S, Puertas MJ (1998) B chromosome meiotic pairing in rye lines of high and low B transmission rate. Cytogenet Cell Genet 81:118-118

Jimenez G, Manzanero S, Puertas MJ (2000) Relationship between pachytene synapsis, metaphase I associations, and transmission of 2B and 4B chromosomes in rye. Genome 43:232-239

Jimenez MM, Romera F, Gonzalez Sanchez M, Puertas MJ (1997) Genetic control of the rate of transmission of rye B chromosomes .3. Male meiosis and gametogenesis. Heredity 78:636-644

Jimenez MM, Romera F, Puertas MJ, Jones RN (1994) B-Chromosomes in inbred lines of rye (*Secale cereale* L). 1. Vigor and fertility. Genetica 92:149-154

[Karafiátová](javascript:;) K, [Bednářová](javascript:;) M, [Said](javascript:;) M, [Čížková](javascript:;) J, [Holušová](javascript:;) K, [Blavet](javascript:;) N, [Bartoš](javascript:;) J (2021) The B chromosome of *Sorghum purpureosericeum* reveals the first pieces of its sequence. *Journal of Experimental Botany* 72: 1606–1616

Kayano H (1957) Cytogenetic studies in *Lilium callosum* III. Preferential segregation of a supernumerary chromosome in EMCs. Proceedings of the Japan Academy 33:553-558

Masonbrink RE, Birchler JA (2010) Sporophytic nondisjunction of the maize B chromosome at high copy numbers. J Genet Genomics 37:79-84

McLeish, J. (1954). B-chromosomes in *Ranunculus ficaria*. Annual Report John Innes Horticultural Institution, 45, 21–22.

Mendelson D, Zohary D (1972) Behaviour and transmission of supernumerary chromosomes *in Aegilops speltoides*. Heredity

Mochizuki A (1957) B chromosomes in *Aegilops mutica* Boiss. Wheat Inf Serv 5:9-11

Müntzing A (1948) Accessory chromosomes in *Poa* *alpina*. Heredity 2:49-61

Neves N, Barao A, Castilho A, Silva M, Morais L, Carvalho V, Viegas W, Jones RN (1992) Influence of DNA methylation on rye B chromosome nondisjunction. Genome 35:650-652

Ohta S (1996) Mechanisms of B-chromosome accumulation in *Aegilops* *mutica* Boiss. Genes Genet Syst 71:23-29

Ortiz M, Puertas MJ, Jimenez MM, Romera F, Jones RN (1996) B-chromosomes in inbred lines of rye (*Secale* *cereale* L). 2. Effects on metaphase I and first pollen mitosis. Genetica 97:65-72

Östergren G (1947) Heterochromatic B‐chromosomes in *Anthoxanthum*. Hereditas 33:261-296

ÖStergren G, Fröst S (1962) Elimination of accessory chromosomes from the roots in *Haplopappus* *gracilis*. Hereditas 48:363-366

Parker J, Jones G, Edgar L, Whitehouse C (1989) The population cytogenetics of *Crepis capillaris*. II. The stability and inheritance of B-chromosomes. Heredity 63:19-27

Pritchard E (1968) A cytogenetic study of supernumerary chromosomes in *Haplopappus* *gracilis*. Can J Genet Cytol 10:928-936

Puertas M, Jimenez G, Manzanero S, Chiavarino AM, Rosato M, Naranjo C, Poggio L (1998a) Genetic control of B chromosome transmission in maize and rye. Cytogenet Cell Genet 81:103-103

Puertas M, Jimenez G, Manzanero S, Chiavarino AM, Rosato M, Naranjo C, Poggio L (2000) Genetic control of B chromosome transmission in maize and rye. Chromosomes Today 13:79-92

Puertas MJ, Gonzalez-Sanchez M, Manzanero S, Romera F, Jimenez MM (1998b) Genetic control of the rate of transmission of rye B chromosomes. IV. Localization of the genes controlling B transmission rate. Heredity 80:209-213

Puertas MJ, Jimenez MM, Romera F, Vega JM, Diez M (1990) Maternal Imprinting Effect on B-Chromosome Transmission in Rye. Heredity 64:197-204

Puteyevsky E, Zohary D (1971) Behaviour and transmission of supernumerary chromosomes in diploid *Dactylis* *glomerata*. Chromosoma 32:135-141

Roman H (1947) Mitotic nondisjunction in the case of interchanges involving the B-type chromosome in maize. Genetics 32:391-409

Romera F, Jimenez MM, Puertas MJ (1991) Genetic control of the rate of transmission of rye B chromosomes .1. Effects in 2B x 0B crosses. Heredity 66:61-65

Ruban A, Schmutzer T, Wu DD, Fuchs J, Boudichevskaia A, Rubtsova M, Pistrick K, Melzer M, Himmelbach A, Schubert V, Scholz U, Houben A (2020) Supernumerary B chromosomes of *Aegilops speltoides* undergo precise elimination in roots early in embryo development. Nat Commun 11:2764

Rutishauser A (1956) Genetics of fragment chromosomes in *Trillium* *grandiflorum*. Heredity 10:195-204

Rutishauser A, Rothlisberger E (1966) Boosting mechanism of B chromosomes in *Crepis capillaris*. Chromosomes today 1:28-30

Semple JC (1972) Behavior of B chromosomes in *Xanthisma* *texanum* DC.: A nonrandom phenomenon. Science 175:666-666

Semple JC (1976) The cytogenetics of *Xanthisma* *texanum* DC. (Asteraceae) and its B‐chromosomes. Am J Bot 63:388-398

Semple JC, Chmielewski JG (1989) Studies on the effects, or lack thereof, of B chromosomes on the morphology and pollen viability in wild and cultivated plants of *Xanthisma* *texanum* (Compositae: Astereae). Canad J Bot 67:1157-1160

Shih-Hsuan Tseng, Shu-Fen Peng, Ya-Ming Cheng (2017) Analysis of B chromosome nondisjunction induced by the r-X1 deficiency in maize. Chrom Res 26:153-162

Su H, Liu Y, Liu Y, Birchler JA, Han F (2018) The behavior of the maize B chromosome and centromere. Genes 9,476

Vosa CG (1962) The transmission of B chromosomes in *Tradescantia* *virginiana*. Chromosome Inf Serv 3:26-28

Wu D, Ruban A, Fuchs J, Macas J, Novak P, Vaio M, Zhou Y, Houben A (2019) Nondisjunction and unequal spindle organization accompany the drive of *Aegilops* *speltoides* B chromosomes. New Phytol 223:1340-1352
